# The low expression of NUP62CL indicates good prognosis and high level of immune infiltration in lung adenocarcinoma

**DOI:** 10.1002/cam4.3877

**Published:** 2021-05-02

**Authors:** Shiqi Ren, Wei Wang, Chenlin Zhang, Yidan Sun, Mengjing Sun, Yingjing Wang, Xiaojing Zhang, Bing Lu, Lanlan Yao

**Affiliations:** ^1^ Department of Clinical Biobank Nantong University Affiliated Hospital Nantong P.R. China; ^2^ Department of Medicine Nantong University Xinling College Nantong P.R. China; ^3^ Department of orthopaedics Qidong Hospital of Chinese Medicine Nantong P.R. China; ^4^ First Teaching Hospital of Tianjin University of Traditional Chinese Medicine Tianjin P.R. China; ^5^ Department of respiration The Six People’s Hospital of Nantong Nantong China

**Keywords:** GEO, immune infiltration, LUAD, NUP62CL, prognostic, TCGA

## Abstract

A primary factor in tumor morbidity and mortality, lung adenocarcinoma (LUAD) is known to be a major subtype of lung cancer, having the lowest survival rate among all other cancers. Using The Cancer Genome Atlas (TCGA) database the relationship between the immune infiltrate and the NUP62CL was explored and the value of the NUP62CL expression in the prognosis and diagnosis LUAD was examined. Using the logistic regression and the Wilcoxon signed‐rank test the relationship between the NUP62CL and the clinico‐pathological features was analyzed. There was a significant correlation between the clinical stage (*p* = 0.005), the N (*p* = 0.004), and the decreased expression of NUP62CL. The prognosis of LUAD with high NUP62CL expression was revealed to be worse than that with low NUP62CL expression (*p* < 0.001) by the Kaplan‐Meier survival analysis. The potentiality of NUP62CL to be a significant factor of prognosis for LUAD was indicated by the analyses of the multivariate and the univariate Cox regression models. In LUAD, the crucial role of recombination and maintenance of telomere as a significant pathway for NUP62CL was suggested by the Gene Set Enrichment Analysis (GSEA). To analyze the correlation between the genes and the tumor infiltrating immune cells the CIBERSORT was used. Moreover the positive correlation with the NUP62CL expression in LUAD of the infiltration level of the tumor infiltrating B lymphocytes and memory CD4+ T cells was exhibited by CIBERSORT. Therefore, NUP62CL may be a new valuable prognostic indicator for LUAD.

## Graphical abstract

High expression of NUP62CL was associated with low OS in patients with LUAD. Cox regression model analysis indicated that NUP62CL was an important prognostic factor of LUAD. Maintaining telomere recombination may be an important signaling pathway for NUP62CL to play a role in LUAD. In addition, NUP62CL was related to the infiltration level of memory CD4+ T cells.

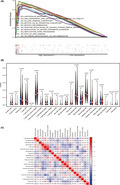

## 1 INTRODUCTION

Lung cancer is a major factor in tumor morbidity and mortality. Rebecca L. et al. established that with 1.8 million patient deaths, denoting almost 18.4% of the total (one in every five), and 2.1 million potential new diagnoses, lung cancer has shown the lowest rate of survival among all cancers.^1^, ^2^ Although the treatment of various therapies has made continuous progress, the 5‐year survival rate is still relatively poor. Lung cancer affords an overall five‐year relative survival of 20.3% in females and 15.5% in males, subsequent to age‐adjustment.^3^ Moreover early diagnosis can significantly improve the prognosis, however, most patients when firstly diagnosis have developed to advanced stage.^2^, ^4^ Hence, the identification of novel immune‐related therapeutic targets and valuable biomarkers for the patients of lung cancer coupled with the further research and development of cancer treatment has become imperative today.

Non‐small cell lung cancer, or NSCLC, with an 80% incidence, is the principal type of lung cancer.^5^ With adenocarcinoma being the leading subtype accounting for nearly 50% of patients afflicted with lung cancer, the primary NSCLC histological phenotypes have been known to include the large cell carcinoma, squamous cell carcinoma, and the adenocarcinoma.^6^ Lung cancer comprises both cancerous and non‐cancerous cells. The knowledge about the role of these non‐cancer cells remains a mystery. The infiltrating immune cells, the vascular cells, and the stromal cells are known to be the components of the non‐cancerous cells in varied proportions. Immune cell infiltration from among these could potentially improve clinical prognosis, and thereby be effectively targeted in therapy, as indicated via recent drug trials that targeted physiological pathways regulating autoimmune responses, as also the duration and severity of disease immune responses, and immune checkpoints, to reveal how they considerably prolonged survival in cases of solid tumors, such as melanoma, NSCLC, renal cell carcinoma and triple negative breast cancer.^7^, ^8^, ^9^, ^10^ In addition, the most widely studied immune cells are tumor infiltrating lymphocytes (TIL). Previous studies have shown that the presence of TIL is potentially predictive and prognostic for specific breast cancer subtypes. As indicated by large‐scale adjuvant studies, in primary biopsy the higher TIL levels have been shown to be associated with reduced recurrence and improved overall survival (OS) particularly in cases with triple negative breast cancer (TNBC), the HER2‐positive, and the human epidermal growth factor receptor 2.^11^, ^12^, ^13^ Amplified TIL levels were also correlated with higher pathological complete response, or PCR rates, subsequent to neoadjuvant therapy in TNBC and HER2‐positive tumor patients.^14^, ^15^, ^16^ Nonetheless, in the case of lung adenocarcinoma the role of these tumor‐infiltrating immune cells remains significantly unknown.

In the mitochondrial transport, DNA repair, gene transcription, and microtubule regulation, the NUP family has been observed to play an important role.^17^, ^18^, ^19^, ^20^, ^21^ The FG repeat domain of NUP98 is encoded by a chimeric gene, the NUP98–NSD1. Approximately 5% of people have t (5; 11) (q35; p15.5) translocation in human acute myeloid leukemia. The study by Gang G. Wang et al. found that the main transformation characteristic of NUP98–NSD1 is that it does not allow cell differentiation, thereby promoting self‐renewal of progenitor cells, and in experiments found that NUP98–NSD1 can enhance self‐renewal by activating Hox‐A gene and Meis1 transcription and to prevent differentiation, the potent leukemia oncogenes HoxA9 and Meis1 are co‐expressed in NUP98–NSD1 immortalized progenitor cells. Acute myeloid leukemia could be caused by NUP98–NSD1.^19^, ^22^ Yumei Leng et al. Showed that tumor protein MUC1‐C was related to human cancer and entered the nucleus through the way of binding with NUP62.^23^ In addition, In the export of HIV retroviral RNA particles in the human cells, the NUP62 has been indicated to play a significant role.^24^ Nonetheless, the involvement of NUP62CL in the development of cancer presently has not been indicated by any study so far.

The prognostic value of the NUP62CL expression in LUAD was evaluated from the data acquired from The Cancer Genome Atlas (TCGA). In the LUAD‐pathogenesis‐linked NUP62CL regulatory network, additional insights into the engagement of the biological pathways were provided by the GSEA. Moreover to study the tumor‐infiltrating immune cells and the NUP62CL correlation, along with the density of the distinct tumor‐infiltrating immune cells under various microenvironments was determined using the CIBERSORT as a recent metagene approach. Moreover, the tumor‐immunity‐interactions correlation, the mechanism involved, besides the understanding and establishing the positive role of the NUP62CL could be possible from the conclusions we drew.

## 2 MATERIALS AND METHODS

### 2.1 Data acquisition and bioinformatics analysis

This study used data from the public domain. The prognostic information of tumors and normal tissues, with the gene expression profiles of the patients of LUAD were downloaded from TCGA (https://portal.gdc.cancer.gov/) for our study, and included 535 tumor tissues. Age, total survival time, gender, clinical stage, grade, TNM stage (T, tumor; N, node; M, metastasis) were included. Subsequently, 503 data along with 522 RNA sequence data were used for further survival analysis and correlation analyses, respectively. In addition, to study the relationship between the invasive immune cells and the expression of NUP62CL the tumor tissue was segregated into high and low expression groups based on the expression of the NUP62CL.

### 2.2 Gene set enrichment analysis

The GSEA computational method helps ascertain statistically significant and consistent variances that exist in any two biological states of an a priori defined set of genes.^25^ In this study, to investigate the potential mechanism of NUP62CL expression in the prognosis of LUAD and to explain the significant differences in the survival rates between the low and high NUP62CL expression groups, the use of GSEA was done. The nominal *p* value and normalized enrichment score (NES) helped assess pathway enrichment (*p* < 0.05, FDR < 0.25).

### 2.3 Evaluation of tumor infiltrating immune cells

As per normalized gene expression data, the relative proportion of 22 kinds of immune cell infiltration was inferred by CIBERSORT algorithm. In other words, deemed to be the smallest depiction of any cell type, the set of reference gene expression values was deployed using the deconvolution algorithm of CIBERSORT. From a large sample of mixed cell types, to infer the proportion of the cell types the Support Vector Regression (SVR) was used based upon these values. Standard annotation files helped compile gene expression datasets, which were then uploaded to the CIBERSORT portal (http://cibersort.stanford.edu/). The algorithm runs with 1000 default signature matrices arranged. CIBERSORT validates conclusions via Monte Carlo sampling, wherein a *p* value is derived to deconvolute each sample. Moreover, CIBERSORT inferred 22 kinds of immune cells. To assess the effect of NUP62CL expression, 535 samples from TCGA were used to represent all genes.

### 2.4 Verification analysis of NUP62CL

From the Gene Expression Omnibus (GEO) repository the GSE13213 data set was downloaded and 117 samples were collected. Using the R survival package, the relationship between NUP62CL expression and the OS was analyzed in the patients with LUAD. To analyze the 9736 tumor and the 8587 normal samples contained in the TCGA and the GTEX, using the standard processing flow, the online database GEPIA was used.^26^ In the patients with LUAD, the relationship between the expression of NUP62CL and the OS was analyzed using GEPIA. The differential expression of NUP62CL under the various pathological stages was compared, while the differential expression of the NUP62CL in the normal and the tumor tissues was observed through boxplot. In addition, the protein locations could be evaluated accurately using the antibody profiles from the 32 human tissues and their protein expression profiles from the Human Protein Atlas network database (HPA) (www.proteinatlas.org). To analyze the expression of NUP62CL in the LUAD and the normal tissues, the HPA was used.

### 2.5 Statistical analysis

R version.3.5.1 assayed all statistical queries. The correlation of the clinico‐pathological features with NUP62CL was evaluated through the logistic regression and the Wilcoxon signed‐rank test. To analyze the impact of the NUP62CL expression on the clinical characteristics of patients with LUAD and their OS, the Cox regression model was applied. All tests were regarded as statistically significant with the *p* < 0.05.

## 3 RESULTS

### 3.1 Patient characteristics

The clinical and gene expression data of 522 tumors, presented in Table 1, were downloaded from TCGA database. In our study, a total of 280 female patients and 242 male patients were analyzed. The disease cited stage wise as a patient count/percentage, was: Stage I: 279/53.45%, Stage II: 124/23.75%, Stage III: 85/16.28%, and Stage IV: 26/4.98%. The tumor included 32.95% T1 (*n* = 172), 53.83% T2 (*n* = 281), 9.00% T3 (*n* = 47), and 3.64% T4 (*n* = 19). The Node included 64.17% N0 (*n* = 335), 18.20% N1 (*n* = 95), 14.37% N2 (*n* = 75), and 0.38% N3 (*n* = 2). A total of 25 cases (4.79%) had distant metastases (Table 1).

**TABLE 1:** Characteristics of LUAD patients in TCGA and GEO database

Clinical characteristics	TCGA (522) %		GEO (117) %	
Age (mean ± SD)	65.33 ± 10.02		60.68 ± 10.17	
Survival time (year)	2.11 ± 2.28		5.38 ± 2.40	
Gender				
Female	280	53.64	57	48.72
Male	242	46.36	60	51.28
Stage				
I	279	53.45	79	67.52
II	124	23.75	13	11.11
III	85	16.28	25	21.37
IV	26	4.98	0	0
T				
1	172	32.95	54	46.15
2	281	53.83	50	42.74
3	47	9	8	6.84
4	19	3.64	5	4.27
M				
0	353	67.62	117	100
1	25	4.79	0	0
N				
0	335	64.17	87	74.36
1	95	18.2	8	6.84
2	75	14.37	22	18.8
3	2	0.38	0	0

Abbreviations: M, metastasis; N, node; T, tumor.

### 3.2 Association with clinico‐pathological variables and NUP62CL expression

**FIGURE 1:**
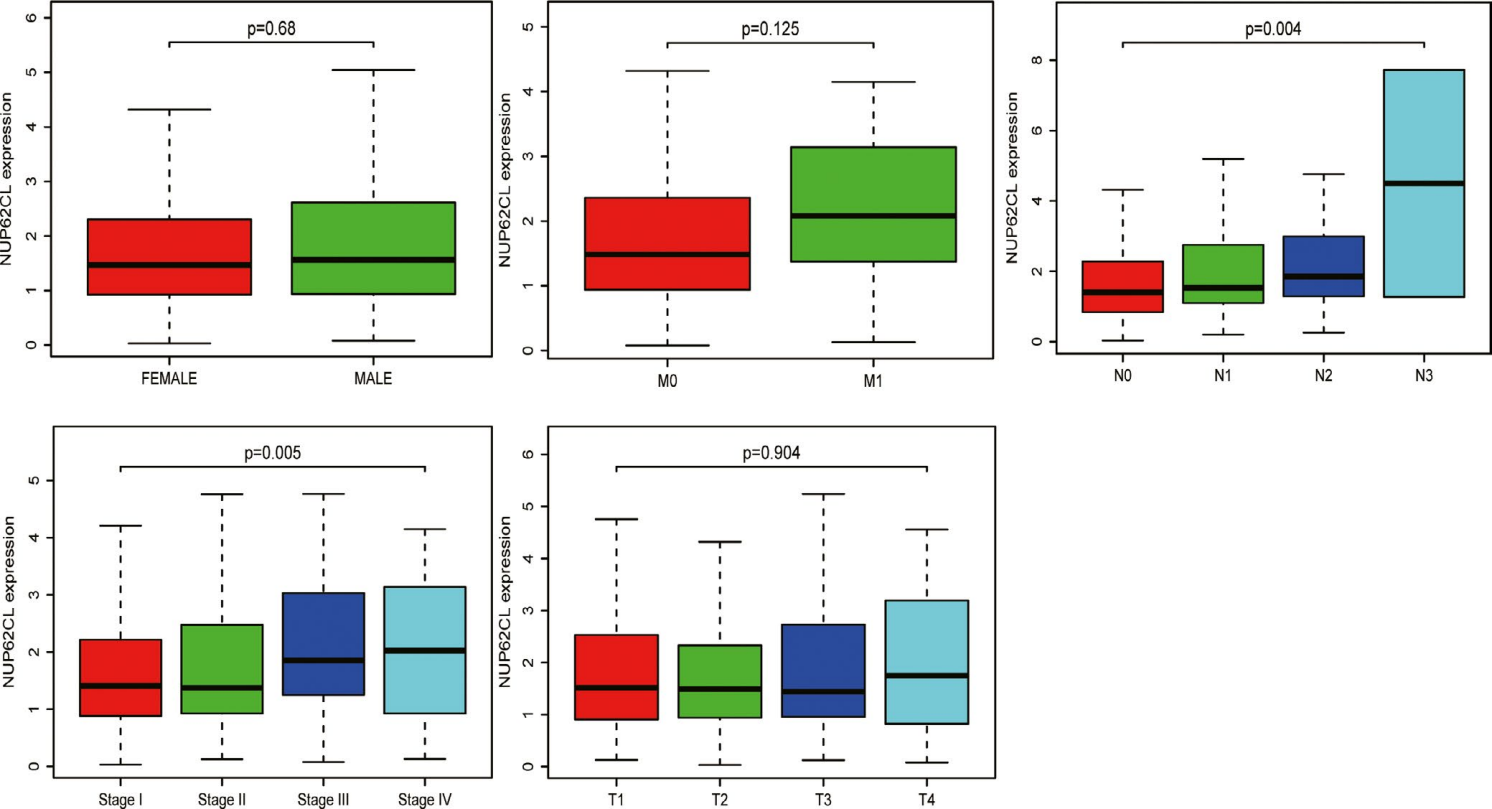
Association with NUP62CL expression and clinicopathologic characteristics, including: gender, clinical stage, and TNM stage

We evaluated NUP62CL expression data across all patient characteristics derived from TCGA. Figure 1 indicated a significant correlation of NUP62CL decreased expression with clinical stage (*p* = 0.005) and N (*p* = 0.004). According to logistic regression analysis, the reduced NUP62CL expression was significantly correlated with gender (OR=0.66, *p* = 0.019), the clinical stage (OR = 0.30, *p* = 0.009, for IV vs. I), T (OR = 0.50, *p* = 0.041, for 3 vs. 1), N (OR = 0.49, *p* = 0.007, for 3 vs. 0) and M (OR = 0.37, *p* = 0.029, for 1 vs. 0) (Table 2). Therefore, LUAD with high NUP62CL expression presents a greater likelihood of progressing to advanced stages and metastasizing distantly, relative to the low expression group.

**TABLE 2:** NUP62CL expression associated with clinical pathological characteristics (logistic regression)

Clinical characteristics	Total (N)	Odds ratio in EMID1 expression	*p*‐value
Gender (male vs. female)	522	0.66 (0.46, 0.93)	0.019
Age (≥65 vs. <65)	522	0.75 (0.52, 1.06)	0.105
Stage (IV. vs. I)	305	0.30 (0.11, 0.71)	0.009
T (3 vs. 1)	219	0.50 (0.25, 0.96)	0.041
M (1 vs. 0)	378	0.37 (0.14, 0.87)	0.029
N (3 vs. 0)	337	0.49 (0.28, 0.81)	0.007

Abbreviations: M, metastasis; N, node; T, tumor.

### 3.3 Survival analysis and multivariate analysis of NUP62CL

**FIGURE 2:**
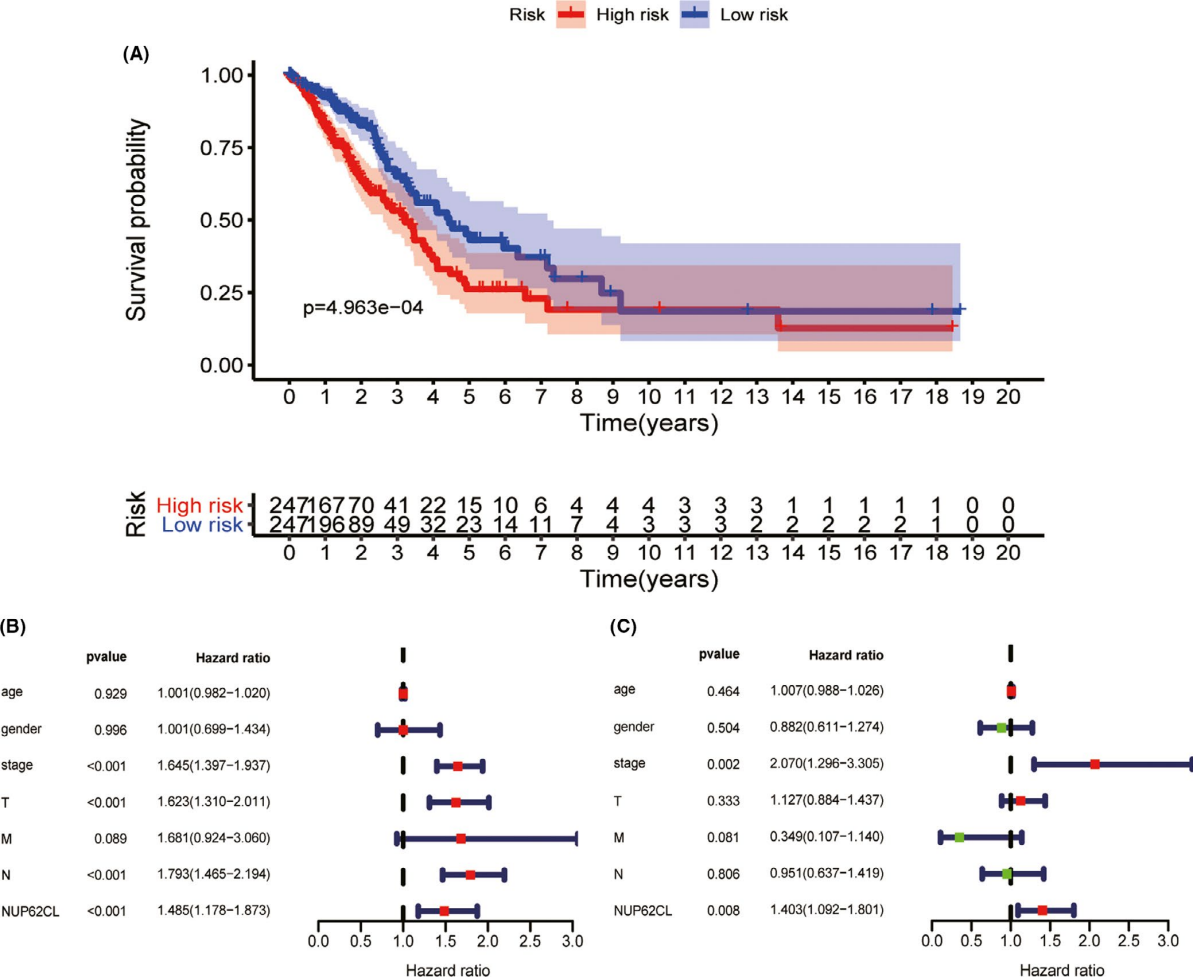
A, Survival analysis of NUP62CL by Kaplan Meier survival curve. B, Univariate analysis of NUP62CL. C, Multivariate survival model of NUP62CL. M, metastasis; N, node; T, tumor

The prognosis of the low expression of NUP62CL was shown to be better than that of the high expression of NUP62CL (*p* < 0.001) by the Kaplan‐Meier survival analysis, as illustrated in Figure 2A. The high expression of NUP62CL having a significant relationship to the low OS of the LUAD patients was indicted by the Univariate analysis. Other clinicopathological variables, including stage, T, N, were also associated with low survival in patients with LUAD. The probability of the NUP62CL being an independent prognostic factor for LUAD was demonstrated by the Multivariate analysis (Table 3, Figure 2B,C).

**TABLE 3:** A, Associations with overall survival and clinicopathologic characteristics in TCGA patients using Cox regression. B, Multivariate survival model after variable selection

Clinicopathologic variable	HR	HR.95L	HR.95H	*p*‐value
(A)				
Age	1.00	0.98	1.02	0.929
Gender	1.00	0.70	1.43	0.996
Stage	1.64	1.40	1.94	<0.001
T	1.62	1.31	2.01	<0.001
M	1.68	0.92	3.06	0.089
N	1.79	1.46	2.19	<0.001
NUP62CL	1.49	1.18	1.87	0.001
(B)				
Stage	2.07	1.30	3.31	0.002
T	1.13	0.88	1.44	0.333
N	0.95	0.64	1.42	0.806
NUP62CL	1.40	1.09	1.80	0.008

Abbreviations: M, metastasis; N, node; T, tumor.

### 3.4 Identification of a NUP62CL linked signaling pathway enabled by GSEA

**FIGURE 3:**
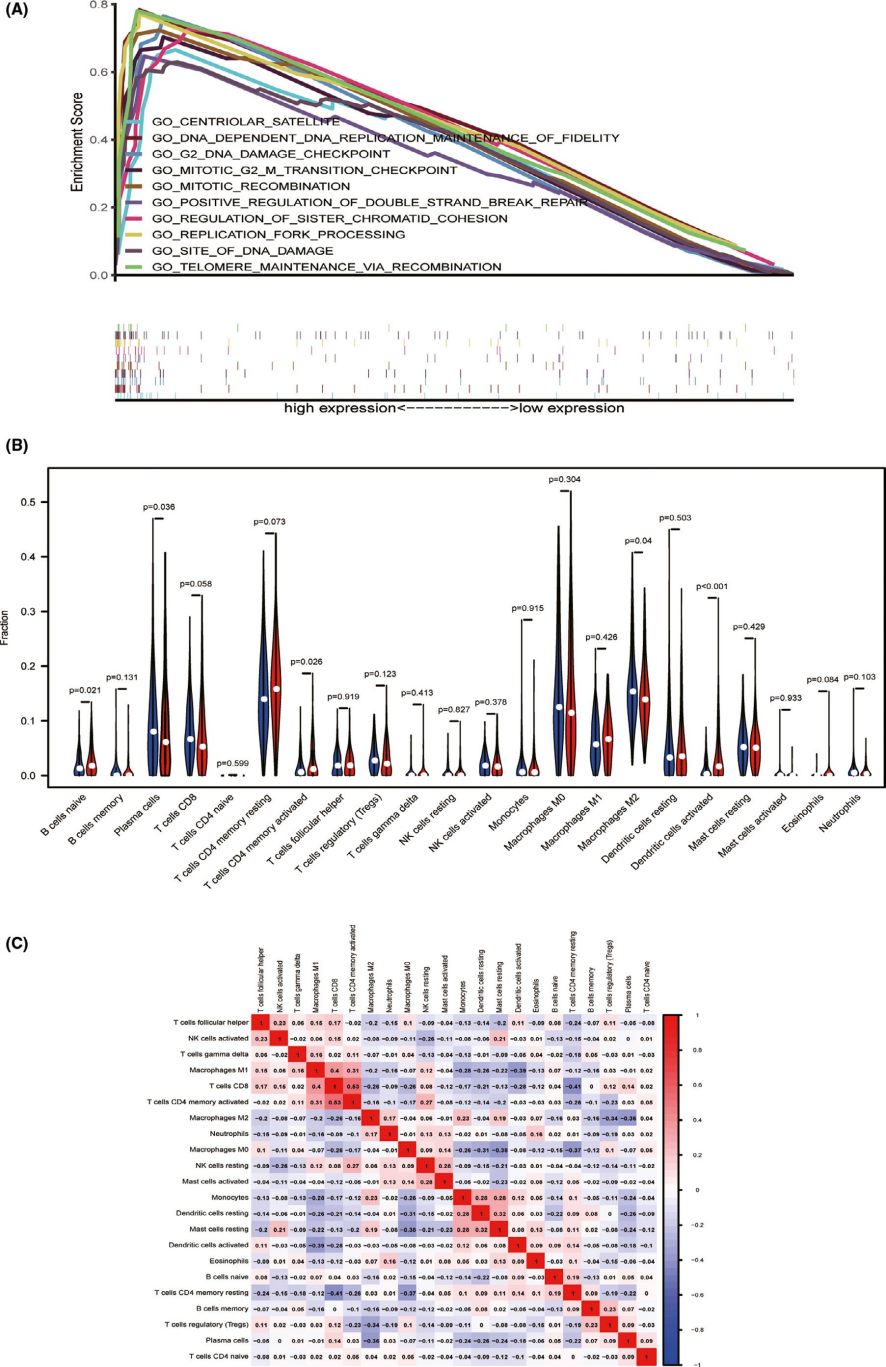
A, GO term analysis showed 10 positive correlation groups. B, The proportion of 22 immune cell subtypes in high and low NUP62CL expression group. Red indicated high expression and blue indicated low expression. C, Heatmap of 22 immune infiltration cells in LUAD samples

The Gene Set Enrichment Analysis (GSEA) between the high and the low expression data of NUP62CL was conducted to identify the significant signaling pathways activated in the Lung adenocarcinoma. Table 4 displays the details of the significant variances (FDR < 0.25, NOM *p* < 0.05) in the enrichment of MSigDB Collection (c5.all.v7.0.symbols.gmt), presented by GSEA. The differentially enriched high expression phenotypes in the NUP62CL have been exhibited in the Figure 3A as, DNA dependent DNA replication maintenance of fidelity, g2 DNA damage checkpoint, replication fork processing, telomere maintenance via recombination, site of DNA damage, positive regulation of double strand break repair, mitotic recombination, regulation of sister chromatid cohesion, centriolar satellite, mitotic g2m transition checkpoint.

**TABLE 4:** Signaling pathways most significantly correlated with NUP62CL expression based on their normalized enrichment score (NES) and *p*‐value

GO name	NES	NOM *p*‐val	FDR *q*‐val
Positive			
DNA dependent DNA replication maintenance of fidelity	2.288	0.000	0.002
G2 DNA damage checkpoint	2.329	0.000	0.003
Replication fork processing	2.257	0.000	0.005
Telomere maintenance via recombination	2.168	0.000	0.033
Site of DNA damage	2.117	0.004	0.069
Positive regulation of double strand break repair	2.102	0.000	0.076
Mitotic recombination	2.091	0.000	0.078
Regulation of sister chromatid cohesion	2.066	0.000	0.090
Centriolar satellite	2.070	0.000	0.097
Mitotic g2m transition checkpoint	2.047	0.002	0.098

Note

Gene sets with NOM *p* < 0.05 and FDR *q*‐val < 0.25 are considered as significant.

Abbreviations: FDR, false discovery rate; NES, normalized enrichment score; NOM, nominal.

### 3.5 Relationship between the tumor‐infiltrating immune cells and the NUP62CL expression

To investigate whether NUP62CL expression is associated with immune infiltration in lung adenocarcinoma, to explore the gene expression profiles of the samples downloaded and to infer the density of the 22 immune cells the CIBERSORT resource was deployed in this study. The total 535 tumor samples were segregated according to the NUP62CL expression into two parts as, 268 samples of high expression and 267 samples of low expression. Subsequently, using CIBERSORT the relative proportion of the 22 kinds of immune cell infiltration in these tumor samples were estimated. The result of the relative proportion of these immune cell infiltration is shown in Figure 3B. In the high expression group, B cells naïve (*p* = 0.021), T cells CD4+ memory activated (*p* = 0.026) and the activated (*p* < 0.001) dendritic cells were found to be significantly increased. However, the Macrophages M2 (*p* = 0.04) and the Plasma cells (*p* = 0.036) were observed to have been significantly increased in the low expression group. Moreover a weak to moderate correlation was presented by the diverse tumor‐infiltrating immune cells subgroups (Figure 3C).

### 3.6 Verification analysis

**FIGURE 4:**
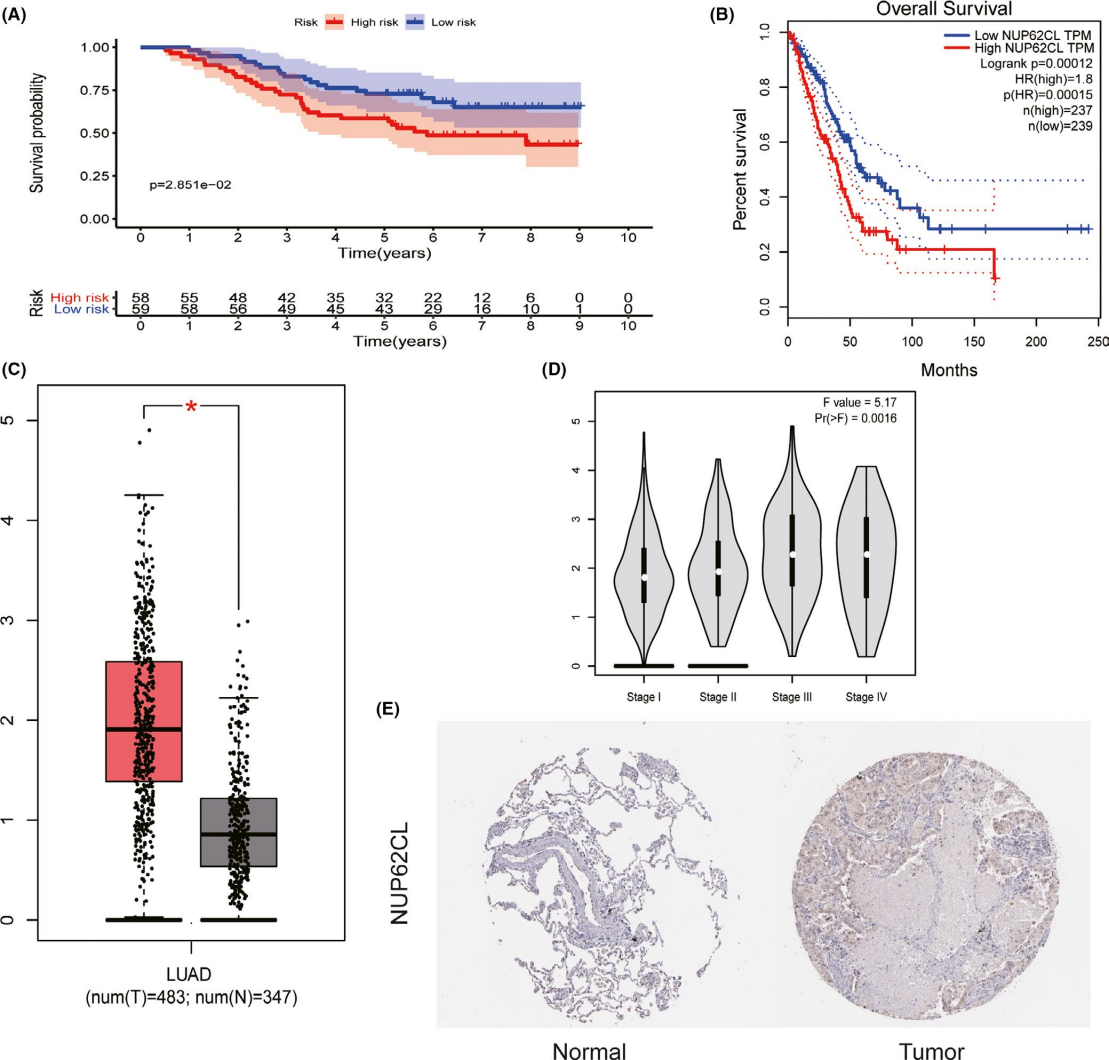
A, The expression level and overall survival of NUP62CL in GEO database. B, The expression level and overall survival of NUP62CL in GEPIA. C, The expression level of NUP62CL in normal and tumor tissues obtained from GEPIA. D, Differential expression of NUP62CL in different pathological stage of GEPIA. E, The expression of NUP62CL protein in lung by HPA immunohistochemistry

To determine whether the prognosis of NUP62CL is reliable, we used GEO database, GEPIA database and HPA database to verify NUP62CL. GSE13213 was downloaded from GEO database and included 117 sample information (Table 1). The high OS in patients with LUAD was found to be associated with the low expression of NUP62CL from the analysis of the data (Figure 4A). From the GEPIA database, it was found that the expression of NUP62CL was significantly higher in the tumor tissues than in the normal group (Figure 4B). Late pathological stage and low OS (*p* < 0.001) were found to be correlated significantly with the high expression of NUP62CL (Figure 4C,D). Higher incidence of the expression of NUP62CL in tumor tissue than in the normal tissue was indicated by the immunohistochemistry analysis from HPA (Figure 4E).

## 4 DISCUSSION

Lung cancer is a leading morbidity and mortality factor, with adenocarcinoma as the primary subtype. In‐depth research on lung adenocarcinoma is imperative to improve the prognosis and the survival rate of the patients with lung cancer.^27^ Possibly, our study might be the first to suggest the association with cancer of the NUP62CL expression and its potential to be a biomarker in the lung adenocarcinoma prognosis.

Our study established an increased NUP62CL expression in LUAD as correlating with overall survival and poor prognosis, as well as advanced clinic‐pathological aspects, such as clinical stage, tumor, node, metastasis. To explore further the functionality of the NUP62CL in LUAD and to specify the following as differentially enriched in its high expression phenotype the GSEA was deployed by the study, DNA‐dependent DNA replication maintenance of fidelity, g2 DNA damage checkpoint, replication fork processing, telomere maintenance via recombination, site of DNA damage, positive regulation of double strand break repair, mitotic recombination, regulation of sister chromatid cohesion, centriolar satellite, mitotic g2m transition checkpoint. Moreover, a significant correlation was established between the immune infiltration levels and the NUP62CL through CIBERSORT in case of the LUAD afflicted patients, including B cells naive, T cells CD4 memory activated, Dendritic cells activated, Plasma cells and Macrophages M2. The potentiality of the NUP62CL to be an independent prognostic biomarker in LUAD was suggested by the results of this study.

Telomerase expression is regulated by receptor cells, so telomere sequence will wear out gradually in each round of DNA replication. Finally, this loss leads to the failure of chromosome ends to distinguish themselves from DNA double strand breaks, and to produce DNA damage reactions that prevent further proliferation of cells. Telomere wear limits the number of times cells enter the replication cycle, and constitutes the internal source of cell death. Therefore, proper telomere function can prevent tumor.^28^ On the contrary, telomere dysfunction can trigger the fusion of chromosome ends, which leads to genomic instability and promotes tumor development.^29^ Moreover, most cancer cells reactivate telomerase, leading to the avoidance of growth arrest and thus infinite proliferation.^28^ Therefore, we speculate that NUP62CL can reactivate telomerase and promote the development of LUAD. However, the mechanism between melanin pathway and lung adenocarcinoma needs further study.

The independent prediction of the survival in cancer and the sentinel lymph node status could be enabled by the tumor infiltrating lymphocyte as a primary prognostic biomarker in the tumor progression.^30^, ^31^ The NUP62CL regulated tumor immunology was conclusively indicated by the positive correlation with memory CD4+ T cells and the significant aspect of our study entailing the NUP62CL expression regarding the immune infiltration levels in LUAD. Cornelia Voigt et al. found that memory CD4+ T cells can produce and release IL‐22 through the regulation of IL‐1 in cancer cells, thus promoting the growth of tumor.^32^ Therefore, we believed that the increased expression of NUP62CL can regulate the production of IL‐22 by memory CD4+ T cells, thus promoting the development of LUAD. The tumor infiltrating B lymphocytes play an important role in the tumor development and exist in all stages of cancer as an indispensable part of the tumor microenvironment. The importance of the B cells infiltrating tumors as regulators of lung cancer progression was indicated by Wang et al.^33^ on one hand, by promoting the T cell response and secreting the tumor‐specific antibodies, the tumor infiltrating B lymphocytes could exert anti‐tumor immunity; on the other hand, B cells can also develop into an immunosuppressive phenotype that secretes IL‐10, thereby promoting tumor development. Our research results showed that highly expressed tumor infiltrating B lymphocytes may be an important factor in promoting the development of LUAD. Previous studies have found that tumor‐infiltrating T cells and B cells are very close, and the functional interaction between them can enhance local immune activation, thereby contributing to a better prognosis for cancer patients.^34^ However, in LUAD, whether tumor‐infiltrating T cells and B cells also have such a role before to promote the development of tumors, this needs more research to explore.

Nonetheless, there were several shortcomings in this study. First, the inevitable missing of certain useful information due to the less number of samples of the clinical data types. Second, the signal mechanism at the cytological level was not analyzed by the study. Nonetheless, an expanded clinical sampling in future research would be needed to validate our conclusions.

To conclude, the mechanism of the NUP62CL, the survival outcomes, and the relationship between clinico‐pathologic variables and the NUP62CL in cases of LUAD were explored and assessed by our study. Telomere maintenance via recombination could potentially constitute the key NUP62CL‐regulated pathways in LUAD. Moreover, the key pathway of NUP62CL regulation in lung adenocarcinoma needs further validation. Overall, with reference to LUAD, we analyzed 22 immune cell subsets and ascertained NUP62CL significantly correlated with tumor‐infiltrating memory CD4+ T cells and tumor infiltrating B lymphocytes, primarily as potential prognostic biomarkers for progression. Therefore, NUP62CL may be a promising prognostic biomarker of lung adenocarcinoma.

## CONFLICTS OF INTEREST

The authors declare that they have no conflict of interest.

## AUTHORS CONTRIBUTIONS

Shiqi Ren and Wei Wang conceived and designed the experiments, performed the experiments, prepared figures and/or tables, and approved the final draft. Chenlin Zhang and Yidan Sun analysed the data, prepared figures and/or tables, and approved the final draft. Mengjing Sun, Yingjing Wang, Xiaojing Zhang performed the experiments, prepared figures and/or tables, and approved the final draft. Ziheng Wang conceived and designed the experiments, authored or reviewed drafts of the paper, and approved the final draft. Bin lu conceived and designed the experiments, analysed the data, and approved the final draft. Lanlan Yao conceived and designed the experiments, analysed the data, authored or reviewed drafts of the paper, and approved the final draft.
